# Acute Idiopathic Scrotal Edema, an Underrecognized Cause of Scrotal Pain in Children: A Case Report and Literature Review

**DOI:** 10.7759/cureus.57988

**Published:** 2024-04-10

**Authors:** Luisa María Benjumea Gómez, Ana Fernanda Muñoz Durán, Brayan Muñoz-Caicedo, Leidy Vanessa Aguirre Carvajal, Johan Sebastian Lopera Valle

**Affiliations:** 1 Department of General Medicine, Sura, Medellín, COL; 2 Department of General Medicine, Fundación Universitaria San Martín, Sabaneta, COL; 3 Department of Radiology, Universidad de Antioquia, Medellín, COL; 4 Department of Ophthalmology, Unión Temporal San Vicente CES, Medellín, COL; 5 Department of Interventional Radiology, San Vicente Fundación, Medellín, COL

**Keywords:** child, testis, ultrasonography, edema, scrotum, acute pain

## Abstract

Acute idiopathic scrotal edema is a clinical entity predominant in children under 10 years whose true incidence is unknown in our setting. Diagnosis is challenging and avoids unnecessary surgeries. We present the case of an idiopathic acute scrotal edema with ultrasound findings highly suggestive of the diagnosis, which was managed conservatively with complete signs and symptoms resolution after discharge. We aim to review the ultrasound characteristics and differential diagnosis of this disorder.

## Introduction

Acute idiopathic scrotal edema (AISE) is a benign cause of acute scrotal edema first described by Qvist in 1956 [1,2]. The exact etiology remains unknown but has been related to hypersensitivity and allergic reactions without identifying the triggering factor [3]. Also, infectious microorganisms, such as beta-hemolytic streptococcus and the Epstein-Barr virus, have been attributed to it [4]. It affects mainly children and adolescents, accounting for about 10% of cases of acute scrotum [1,5].

AISE is characterized by sudden and primarily unilateral scrotal edema with variable degrees of pain and no alterations in the laboratory tests [3,6]. The diagnosis is based on a judicious physical examination; however, it sometimes poses a diagnostic challenge with differential diagnoses like testicular torsion, so detecting AISE can prevent unnecessary surgical explorations [1,7,8]. The imaging of choice is testicular Doppler, essential to rule out surgical or infectious diagnoses and looking for the "fountain sign", highly suggestive of AISE, which can be treated conservatively with edema resolving in one to three days [7,9,10].

We present a case of AISE in a 10-year-old boy.

## Case presentation

A 10-year-old male patient with a past medical history of asthma consulted the emergency service for three days of progressive discomfort in the right testicle that was associated with mild pain and erythema. There was no trauma, sting, fever, nausea, vomiting, extreme pain, or other associated symptoms. On physical examination, the only abnormal finding was a right-sided scrotal erythema and edema with pain on palpation. A Doppler ultrasound was requested with a clinical suspicion of testicular torsion that was ruled out.

**Figure 1:**
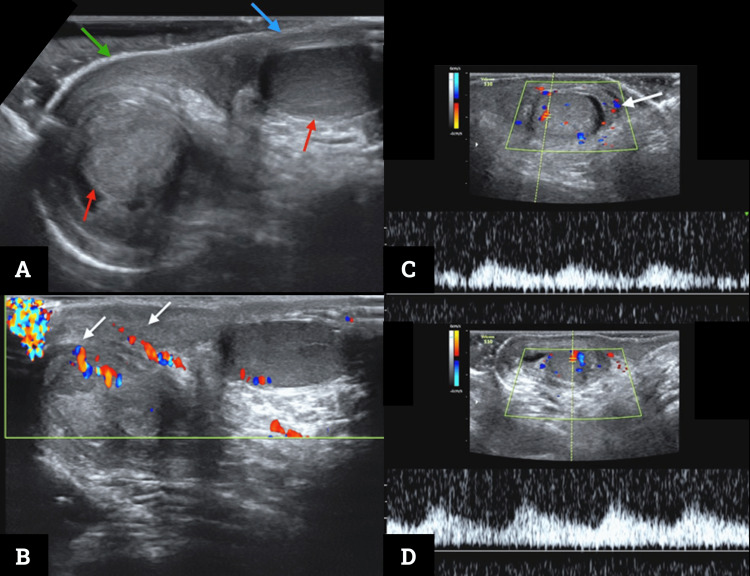
Testicular Doppler ultrasound (A) B-mode examination transverse plane in the scrotum with both testes in the scrotal sacs (red arrows). Asymmetrical thickening of the scrotum on the right side (green arrow) compared to its contralateral (blue arrow). (B) Color Doppler ultrasound oblique plane through the scrotum. Increased color Doppler signal in the right scrotum (white arrows) secondary to hyperemia and configuring the “fountain sign.” (C) and (D) Color and spectral Doppler ultrasound in the longitudinal plane to the right and left testis, respectively. Spectral analysis shows symmetrical and normal low resistance waves, typical of standard testicular circulation—the increased color Doppler signal from the right scrotum is persistent (white arrow in panel C).

In this scenario, a diagnostic impression of epididymal orchitis was in question, and laboratory tests were performed. They showed only mild eosinophilia, and the quantitative ultrasensitive C-reactive protein was less than 0.50 mg/dL (reference value 0-0.6 mg/dL). Given these findings, a new Doppler testicular ultrasound was performed to look for differential diagnoses (Figure 1).

The new ultrasound confirmed that there were no signs of testicular torsion and suggested the possibility of idiopathic scrotal edema supported by the fountain sign. Under these considerations, the patient and his family were reassured of a likely benign and transitory AISE managed with supportive anti-inflammatory medications, and he was discharged. In the follow-up consultation, the patient reported that his symptoms had resolved three days after the discharge and remained asymptomatic, and the physical examination was normal, requiring no additional management.

## Discussion

AISE is a self-limiting cause of acute scrotal edema that happens mainly in children [1]. It represents a diagnostic challenge that prevents surgical explorations [7]. It was described for the first time in Sweden by Qvist in 1956, reporting a prevalence of 20% of acute scrotal edema in children in his publication [2]. However, other series describe AISE as the most frequent cause of acute scrotal edema in children under 10 years of age, reaching a prevalence of up to 69% [1,11].

The exact etiology remains unknown and has been related to multiple theories such as infection by the Epstein Barr virus or a hypersensitivity reaction related to a variant of angioneurotic edema, which would explain the eosinophilia demonstrated in up to 66.7% of cases (as in our case) [1,3,4,7].

AISE is characterized by sudden scrotal edema and erythema that can extend to the perineum, mostly unilateral, with variable pain intensity [6]. Patients are stable and generally looking good. There is no distress, fever, discomfort, elevation of inflammatory markers, or alterations in the urinalysis [3]. It lacks response to antibiotics and improves with antihistamines [1].

Differential diagnoses include testicular torsion, torsion of the testicular appendage, epididymal-orchitis, scrotal trauma, incarcerated inguinal hernia, testicular tumors with internal hemorrhage, cellulitis, Henoch Schölein vasculitis, and Fournier's gangrene [12,13].

The best imaging modality is testicular Doppler ultrasound, with the role of confirming or excluding the leading causes of the acute scrotum, avoiding unnecessary surgical explorations [1,7]. The main ultrasound findings are edema with increased thickness and echogenicity of the scrotal wall, associated with a pattern of hypervascularization, which is irrigation from the internal and external pudendal arteries branches arising from the anterior and posterior walls of the scrotum, which gives the described “fountain sign”, typical of this entity [7,8,10,14,15]. Other relevant findings include testes of normal shape, reactive hydrocele, and increased size with hypervascularization of lymph nodes [2]. Computed tomography and magnetic resonance imaging are left only for selected cases due to radiation and high cost or availability [14].

Once the diagnosis is established, the natural history and prognosis are excellent without sequelae [7]. Symptoms last 6 to 72 hours while edema and scrotal erythema disappear after 48 hours with no residual changes. In 21% of cases, AISE may recur 1 to 3 times without complications [1,5,7,10].

Finally, our case report is important because it illustrates how the imaging appearance of the asymmetric “fountain sign” and edematous scrotum, in the absence of infectious, inflammatory, and surgical causes, was the leading point in considering the diagnosis of AISE. It then remarks on the radiologist's opportunity to enhance multidisciplinary work in the hospital setting, where correct imaging interpretation sheds light on clinical scenarios.

## Conclusions

AISE is a self-limited cause of acute scrotal edema that is unsuspected in many cases. The ultrasound findings are essential to rule out other critical or surgical diagnoses. When considering this entity, the scrotal edema and the “fountain sign” are important hints, aiming to avoid unnecessary surgical explorations and shift management toward ruling out differential diagnoses, conservative interventions, and follow-up.
